# Severity-associated cross-reactive anti-sarbecovirus antibody responses in COVID-19 convalescents and isolation of a dual-targeting monoclonal antibody with cross-neutralizing activity

**DOI:** 10.3389/fimmu.2026.1839618

**Published:** 2026-06-15

**Authors:** Qian Wu, Ling Niu, Yueyang Zhang, Tan Tan, Yuchen Yang, Shuang Li, Bo Liu, Jun Chen, Chun Huang, Qijie Wang, Yabin Hu

**Affiliations:** 1 Translational Medicine Institute & Neurological Medicine Institute, The First People’s Hospital of Chenzhou, The First Clinical College, Xiangnan University, Chenzhou, China; 2 Department of Laboratory Medicine, the Third Affiliated Hospital of Sun Yat-sen University, Guangzhou, China; 3 Biology Teaching & Research Group, Linyi Middle School of Chenzhou, Chenzhou, China; 4 Department of Infectious Disease, The Central Hospital of Shaoyang, Shaoyang, China

**Keywords:** cross-neutralizing, cross-reactive antibodies, disease severity, dual-targeting antibodies, sarbecovirus

## Abstract

**Background:**

Sarbecoviruses pose a persistent pandemic threat due to their zoonotic potential and high recombination capacity. This study investigated how disease severity influences cross-reactive antibody responses and the isolation of cross-neutralizing monoclonal antibodies.

**Methods:**

Plasma samples from 67 COVID-19 convalescents (severe, n=17; non-severe, n=50) were compared for cross-reactive antibody responses against seven sarbecovirus spike proteins. Memory B cells were sorted using dual S1/S2 fluorescent probes to isolate monoclonal antibodies.

**Results:**

Severe convalescents exhibited significantly higher neutralization titers against six animal-derived sarbecoviruses, responses that persisted for up to one year and were further enhanced by vaccination. From dual-targeting B cells, we isolated mAb 1D6, which simultaneously engages the S1 (ACE2-blocking) and S2 (fusion-inhibiting) domains. mAb 1D6 neutralized SARS-CoV-2 (IC50 = 5.708 μg/mL) and pangolin-CoV-GD (IC50 = 14.02 μg/mL). In contrast, antibody 1F2 bound both S1 and S2 across all tested sarbecoviruses but lacked neutralizing activity.

**Conclusions:**

Severe COVID-19 was associated with broader and more durable cross-sarbecovirus antibody responses. The dual-targeting antibody 1D6 provides proof-of-concept for a cross-neutralization strategy, although its modest potency requires further optimization for therapeutic application.

## Introduction

The twenty-first century has witnessed three major zoonotic coronavirus outbreaks, each causing a significant global health crisis: the severe acute respiratory syndrome coronavirus (SARS-CoV) outbreak of 2002-2003, the Middle East respiratory syndrome coronavirus (MERS-CoV) outbreak identified in 2012, and most recently, SARS-CoV-2, which emerged in late 2019 and caused the COVID-19 pandemic. These viruses share common evolutionary origins, with phylogenetic evidence indicating bats as their primary reservoir and subsequent spillover to humans via intermediate hosts (1). Recent surveillance studies have revealed the continued circulation of diverse sarbecoviruses (the subgenus of betacoronaviruses that includes SARS-CoV and SARS-CoV-2) in wildlife populations, some of which possess the capability to utilize human ACE2 for cellular entry (2–4). The geographic overlap of different sarbecovirus hosts and the high recombination potential of coronaviruses represent a persistent pandemic threat, necessitating the development of broad-spectrum interventions (5, 6).

The sarbecovirus spike (S) glycoprotein mediates viral entry through binding to host cell receptors and subsequent membrane fusion, making it the primary target for neutralizing antibodies and vaccine development (7). The S protein consists of two functional subunits: the S1 subunit, which contains the receptor-binding domain (RBD) responsible for ACE2 recognition, and the S2 subunit, which mediates membrane fusion through conformational changes (8–10). The structural organization of the spike protein itself offers opportunities for broad-spectrum protection strategies. While the S1 subunit, particularly the RBD, exhibits higher variability among sarbecoviruses, the S2 subunit demonstrates greater structural conservation across the lineage. This differential conservation pattern suggests that simultaneously targeting both subunits could provide broader protection while reducing the likelihood of escape mutations. Recent studies have explored antibodies that simultaneously engage both S1 and S2 domains, providing evidence that such dual-targeting approaches may stabilize the prefusion conformation and prevent viral entry through multiple mechanisms (11).

The relationship between disease severity and the quality of immune responses has emerged as a critical factor in understanding long-term protection. Studies of SARS-CoV-2 convalescents have demonstrated that severe disease is associated with more potent neutralizing antibody responses compared to non-severe cases (12). However, the extent to which disease severity influences the development of cross-reactive immunity against diverse sarbecoviruses remains poorly understood. Furthermore, the durability and breadth of such cross-reactive responses in relation to disease severity have not been comprehensively characterized. In this study, we systematically compared cross-reactive anti-sarbecovirus antibody responses between severe and non-severe COVID-19 convalescents and utilized dual S1/S2 antigen probes to isolate cross-neutralizing monoclonal antibodies. Our approach aimed to understand how natural infection history shapes cross-protective immunity and to identify antibody strategies that could inform the development of broadly effective countermeasures against current and future sarbecovirus threats.

## Materials and methods

### Study subjects

This study analyzed data from 92 participants across three cohorts: (i) 67 COVID-19 recovered patients assessed at 2 months post-infection; (ii) 25 patients evaluated at 12 months post-infection; (iii) the same 25 patients followed at 24 months post-infection after receiving an inactivated SARS-CoV-2 vaccine. COVID−19 diagnosis was based on the WHO interim guidance (2020). Data from the 2-month cohort have been previously published (12). Of the 25 participants followed across 12- and 24-month, 21 were previously reported (13), with 4 additional newly enrolled patients added to the current analysis. Complete clinical data for all patients are provided in **Supplementary Tables 1** and **S2**. All participants were recruited from Shaoyang, Hunan Province, China. The study was approved by the Ethics Committee of Shaoyang Central Hospital (approval no. 2022-043). Written informed consent was obtained from every participant. Peripheral blood (10 mL) was collected in EDTA-coated tubes. Peripheral blood mononuclear cells (PBMCs) and plasma were isolated using lymphocyte separation medium (Stemcell Technologies, Vancouver, Canada), aliquoted, and cryopreserved in liquid nitrogen (PBMCs) or -80 °C freezer (plasma).

### Cell lines

Human embryonic kidney freestyle 293 cells (293F), HEK293-T and Sf9 cells were obtained from ATCC. HEK293-T stably expressing human ACE2 (293T-ACE2) was generated in our laboratory by lentiviral transduction (14). 293F was maintained in FreeStyle 293 medium (Thermo Fisher Scientific, Waltham, USA) at 37 °C with 8% CO_2_ under 125-rpm shaking. Sf9 was cultured statically in Sf-900 II SFM (Thermo Fisher Scientific, Waltham, USA) at 27 °C. HEK293-T and 293T-ACE2 were grown in DMEM supplemented with 10% fetal bovine serum (FBS; OPCEL, Hohhot, China), and 1% penicillin-streptomycin at 37 °C with 5% CO_2_. PBMCs were rested overnight in RPMI-1640 containing 10% FBS prior to single-cell sorting.

### Expression and purification of Sarbecovirus spike S1 and S2

Recombinant spike extracellular domain (S-ECD) proteins from multiple coronaviruses and their subunits were purchased from Sino Biological. These included S-ECDs from (i) SARS-CoV-2 (prototype strain and variants Alpha, Beta, Gamma, Delta, and Omicron BA.1), (ii) SARS-CoV and MERS-CoV, (iii) the human coronaviruses HKU1, OC43, NL63, and 229E, as well as (iv) the SARS-CoV-2 S1 subunit, S2 subunit, and receptor-binding domain (RBD). The corresponding catalog numbers are as follows: 40589-V08B1, 40634-V08B, 40069-V08B, 40606-V08B, 40604-V08B, 40605-V08B, 40589-V08B6, 40589-V08B11, 40589-V08B10, 40589-V08B16, 40589-V08B33, 40591-V08H, 40590-V08B, and 40592-V08B, respectively. Codon-optimized genes encoding the spike S1 or S2 subunits of various sarbecoviruses were synthesized by GenScript (Nanjing, China). These included SARS-CoV (Y463060.1); bat coronaviruses RaTG13 (MN996532.2) and WIV1 (KC881007.1); pangolin coronaviruses PCoV-GX (MT040335.1) and PCoV-GD (MT799524.1); as well as civet coronaviruses SZ3 (AY304486.1) and Civet007 (AY572034.1). The synthesized genes were then cloned into the pcDNA3.1-Strep-tag II vector (for S1) or the pQBD-Strep-tag II vector (for S2). For S1 production, 293F cells were transfected with pcDNA3.1-S1 constructs using polyethylenimine (PEI, Polysciences, Pennsylvania, USA) with supernatants harvested at day 6 post-transfection. Clarification was performed by centrifugation (4,000×g, 20 min), followed by 0.22-μm filtration and purification via Strep-Tactin^®^ Superflow^®^ high-capacity resin (IBA Lifesciences, Göttingen, Germany) on an ÄKTA pure™ system (Cytiva, Marlborough, USA). For S2 production, recombinant baculoviruses (P0) were generated by co-transfecting Sf9 cells with pQBD-S2 and BacIII-G DNA (15). P0-amplified viruses were used to infect fresh Sf9 cells for 72 h, followed by identical clarification and purification procedures as for S1.

### Preparation of fluorescent B-cell probes and single-cell sorting

Prototype SARS-CoV-2 S1 or S2 proteins (1 mg/mL) were labeled with Alexa Fluor 488 or Alexa Fluor 647 (Invitrogen, California, USA) following manufacturer protocols. Cryopreserved PBMCs from three convalescent who had subsequently received an inactivated vaccine (convalescent vaccinees) were thawed. The cells were then rested overnight in RPMI-1640 with 10% FBS. Subsequently, they were washed twice with PBS containing 2% FBS, and blocked with anti-ACE2 monoclonal antibody (5 µg/mL, Sino Biological, Beijing, China) for 30 min at 4 °C. Cells were then incubated with LIVE/DEAD, anti-CD3 antibody, anti-CD19 antibody, anti-CD27 antibody, anti-IgG antibody, S1-AF488 and S2-AF647 for 30 min at 4 °C in the dark (**Table 1**). Single-cell sorting of LIVE/DEAD^-^ CD3^-^ CD19^+^ CD27^+^ IgG^+^ S1^+^ S2^+^ memory B cells was performed using a Beckman MoFlo XDP sorter, with cells deposited directly into individual wells of 96-well plates. Each well contained 7 µL lysis buffer (10% IGEPAL CA-630 (Sigma-Aldrich, St. Louis, USA), 300 ng μL^-^¹ Random Primer (Thermo Fisher Scientific, Waltham, USA), 40 U μL^-^¹ RNAsin (Thermo Fisher Scientific, Waltham, USA), 100 mM DTT (Thermo Fisher Scientific, Waltham, USA)), respectively.

**Table 1:** Flow cytometry antibody panel for single-cell sorting.

Target	Clone	Conjugate	Catalog number	Dilution	Vendor
anti-CD3	OKT3	PE	12-0037-42	1:50	Thermo Fisher Scientific
anti-CD19	HIB19	BV421	562440	1:50	BD Biosciences
anti-IgG	G18-145	PE-CF594	562538	1:25	BD Biosciences
anti-CD27	M-T271	PE-Cy7	356412	1:25	BioLegend
S1-AF488	–	Alexa Fluor™ 488	A20181	0.25 μM	Thermo Fisher Scientific
S2-AF647	–	Alexa Fluor™ 647	A20186	0.125 μM	Thermo Fisher Scientific
LIVE/DEAD	–	–	L34966	1:1000	Thermo Fisher Scientific

### Antibody cloning, expression and purification

Reverse transcription was performed using SuperScript IV (Thermo Fisher, Waltham, USA), and variable heavy (VH) and light-chain (Vκ/Vλ) regions were amplified separately by nested multiplex PCR using IgG heavy chain-specific primers and IgG light chain (kappa/lambda)-specific primers, respectively, as previously described (16). The PCR products were purified and sequenced to determine the H and κ/λ chain sequences. Validated VH/VL pairs were synthesized, and cloned into immunoglobulin expression vectors (AbVec2.0-IGHG1, AbVec1.1-IGKC or AbVec1.1-IGLC2). These specific vectors were obtained as laboratory stocks, and their sequences have been confirmed by sequencing to match those of the original plasmids (Addgene #80795, #80796 and #99575, respectively) described by Wardemann et al. (16). These plasmids were co-transfected into 293F cells at a 1:2 heavy: light chain ratio using PEI. After 5 days, supernatants were filtered through 0.22 μm membranes, and IgG was purified using HiTrap Protein A columns (Cytiva, Marlborough, USA).

### ELISA

High-binding 96-well plates (JETBIOFIL, Guangdong, China) were coated with recombinant full-length spike, RBD, S1 or S2 proteins (200 ng/well; derived from human coronaviruses or sarbecoviruses) in PBS overnight at 4 °C. After five washes with PBS containing 0.05% Tween-20 (PBS-T), plates were blocked with PBS-T supplemented with 2% BSA and 2% FBS for 2 h at 25 °C. Serially diluted plasma samples (2-fold dilutions starting at 1:400) or monoclonal antibodies (1 μg/mL) were added in triplicate and incubated for 1 h at 25 °C. Following washes, horseradish peroxidase (HRP)-conjugated goat anti-human IgG (1:5,000; Jackson ImmunoResearch, PA, USA) was added for 1 h at 37 °C. After final washes, 3,3’,5,5’-tetramethylbenzidine (TMB; Thermo Fisher Scientific, Waltham, USA) substrate was developed for 5–10 min at 25 °C, and reactions were stopped with 1 M H_2_SO_4_. Optical density at 450 nm (OD_450_) was measured using a Varioskan Flash microplate reader (Thermo Fisher Scientific, Waltham, USA). Samples demonstrating OD_450_ values exceeding threefold the mean of negative controls were defined as seropositive. Positive controls (Pos Ctrl) included the previously reported broad neutralizing antibody SCM13-65 (for S1 and RBD) (13) and the broad binding antibody VSM7-75 (for full-length spike and S2) (17). The negative control (Neg Ctrl) was an anti-HCV mAb, HNC-5.

### Competition ELISA

The competitive enzyme-linked immunosorbent assay (ELISA) was performed as previously described with minor modifications (17). In brief, 96-well plates were coated overnight at 4 °C with recombinant SARS-CoV-2 receptor-binding domain (RBD), and then blocked with blocking buffer at 25 °C for 2 h. Competitor monoclonal antibodies (mAbs) were added at a concentration of 10  μg/mL in a volume of 30  μL per well and incubated at 37 °C for 30  min. Subsequently, biotinylated human ACE2 (Sino Biological, Beijing, China) was added to a final concentration of 1 μg/mL in a total volume of 120 μL per well, after which the plates were incubated at 37  °C for 1  h. Following five washes with PBS containing 0.05% Tween-20 (PBST), horseradish peroxidase (HRP)-conjugated streptavidin (Solarbio Life Science, Beijing, China) diluted 1:2000 was added and incubated at 37  °C for 1 h. After a final wash step, substrate solution was added, and absorbance was measured at 450  nm. The percentage of reduction in binding was calculated using the equation:


$$1\;–\;\left(OD_{with\;competitor\;antibody}–\;OD_{blank}\right)/\left(OD_{without\;competitor\;antibody}–\;OD_{blank}\right)\;\times \;100\%$$

### Pseudovirus neutralisation assay

Sarbecovirus spike-pseudotyped viruses were generated by co-transfecting 293T cells with a pcDNA3.1 plasmid encoding the full-length spike protein and the env-deficient HIV-1 backbone vector pNL4-3-Luc-R-E- ΔEnv (kindly provided by Prof. Yi-ping Li, Sun Yat-sen University) at a 1:3 ratio using polyethylenimine (PEI). To enhance infection efficiency, the D501N mutation was introduced into the RaTG13 spike construct; all other animal sarbecovirus spikes were used as wild-type sequences. Supernatants were harvested 48 h post-transfection, clarified (4000×g, 15 min), filtered through 0.22-μm membranes, and stored at -80 °C. For neutralization assays, 293T-ACE2 cells were seeded at 1×10^4^ cells per well in 96-well plates. Plasma or monoclonal antibodies were 3-fold serially diluted (starting at 1:30 for plasma, 10 µg/mL for mAbs) and pre-incubated with pseudovirus for 1 h at 37 °C. The broad neutralizing antibody SCM13–65 and the anti-HCV mAb HNC-5 served as positive and negative controls, respectively. Subsequently, the mixture was added to cells and spin-inoculated (800×g, 30 min). After 48 hours of incubation, luciferase activity was measured in relative light units (RLU) using the Luciferase Assay System (Promega) on a Varioskan Flash Multimode Reader (Thermo Fisher Scientific). Virus-only (VC) and cell-only (NC) controls were included. The percentage of neutralization was calculated using the following equation:


$$\%\;Neutralization=100\;\times \;(1-\frac{RLU(tested\;samples)-RLU(NC)}{RLU(VC)-RLU(NC)})$$

### Structure modeling and docking of antibody–spike protein complexes

The antigen-binding fragments (Fabs) of monoclonal antibodies 1D6 and 1F2 were modeled with AlphaFold 3 in multimer mode using paired heavy- and light-chain sequences. For docking, the trimeric spike ectodomain (PDB:7DWZ) and the RBD (PDB:6M0J) were treated as monomeric units extracted from their respective trimeric assemblies; consequently, these models do not capture the steric constraints or epitope accessibility of the native prefusion trimer. Protein–protein docking between each Fab and the spike protein was performed using the HDOCK server for global, blind docking. For each complex, the top 100 scoring poses were retrieved. These poses were prioritized based on HDOCK score, absence of steric clashes, and engagement of CDR loops with the spike protein surface. Interface residues were identified using the InterfaceResidues script (PyMOLWiki) within PyMOL v2.3.2, employing a 4.0 Å heavy-atom distance cutoff. All structural figures and contact maps were generated using PyMOL v2.3.2.

### Statistical analysis

Normality was assessed using the Kolmogorov-Smirnov test prior to comparative analyses. Spearman’s rank correlation coefficient determined associations between neutralization titers and neutralization breadth. Mann-Whitney U tests evaluated differences between groups. All analyses used GraphPad Prism v10.0 and SPSS v26, with statistical significance defined as P< 0.05 (*), P< 0.01 (**), P< 0.001 (***), P< 0.0001 (****); ns = not significant.

## Results

### Cross-reactive antibody responses are influenced by disease severity

To investigate whether disease severity is associated with cross-reactive antibody responses, we analyzed plasma samples from 67 COVID-19 convalescents stratified by clinical presentation (severe: n=17; non-severe: n=50). As previously reported, severe convalescents exhibited significantly elevated SARS-CoV-2-specific anti-spike IgG titers and pseudovirus neutralization capacity compared to non-severe cases (12). Building upon these findings, we extended our analysis to characterize cross-reactivity against diverse sarbecovirus spike proteins.

**Figure 1:**
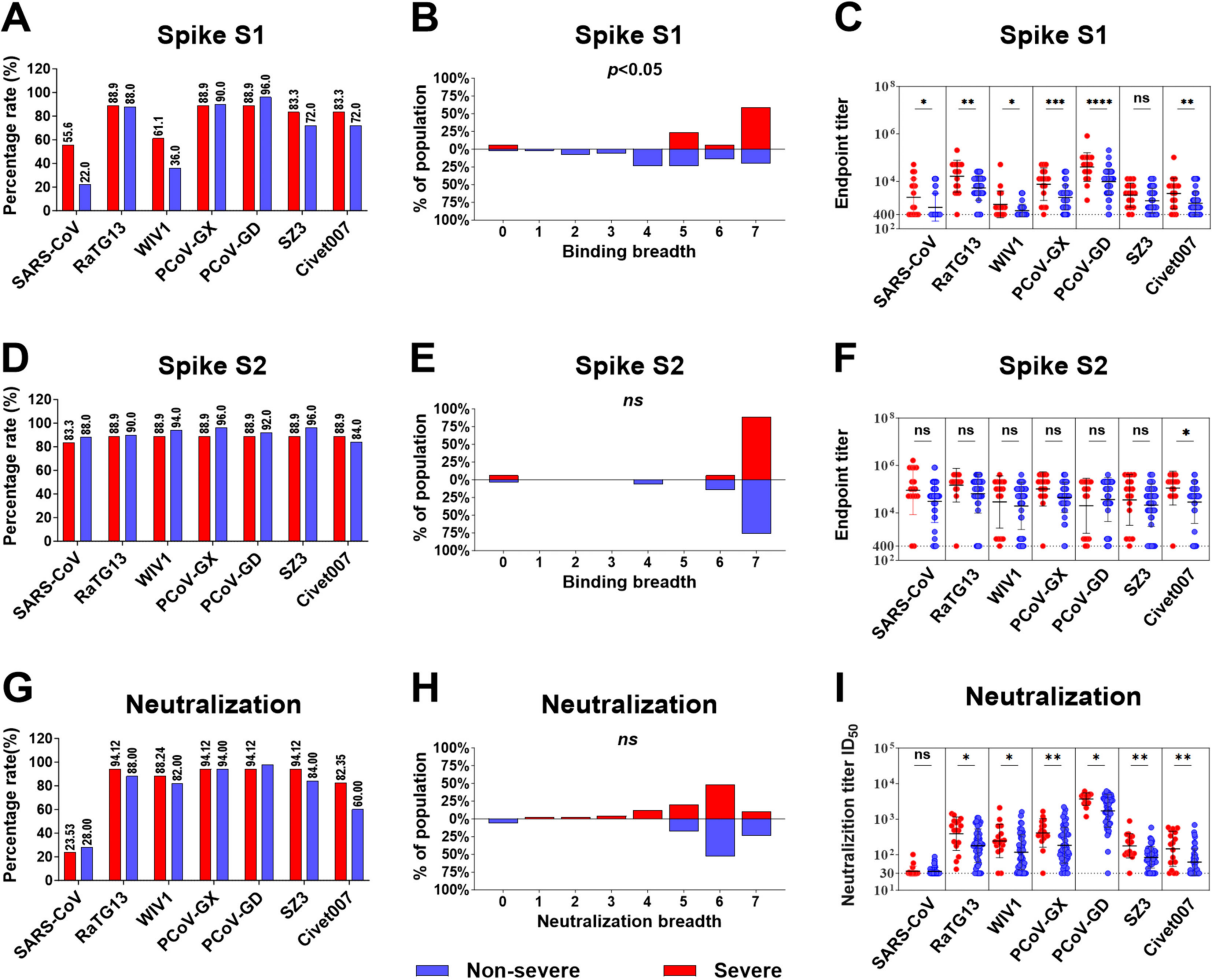
Disease severity-dependent cross-sarbecovirus antibody responses. Cross-reactive antibody responses were systematically compared between severe (n=17) and non-severe (n=50) COVID-19 convalescents against seven sarbecovirus spike subunits. **(A, D)** Seropositivity rates for S1 and S2 subunit binding, respectively. **(B, E)** Binding breadth scores representing the number of viruses recognized (scale 0-7). **(C, F)** Endpoint IgG titers for S1 and S2 subunit binding, respectively. Statistical significance was determined using the Mann-Whitney U test. **(G–I)** Cross-neutralization profiles against sarbecovirus pseudoviruses. **(G)** Neutralization seropositivity rates. **(H)** Neutralization breadth scores representing the number of viruses neutralized (scale 0-7). **(I)** Neutralization titers. Horizontal bars represent median values. Statistical significance was determined using the Mann-Whitney U test. *P< 0.05; **P< 0.01; ***P< 0.001; ****P< 0.0001. ns, not significant.

Employing ELISA-based binding assays, we systematically evaluated IgG responses against the S1 and S2 subunits of seven sarbecoviruses: SARS-CoV, RaTG13, WIV1, PCoV-GX, PCoV-GD, SZ3, and Civet007. Analysis of S1 subunit reactivity showed similar seropositivity frequencies in the severe and non-severe groups (**Figure 1A**). The positive rate for most S1 subunits in the severe group ranged from 88.3% to 88.9%, with exceptions for SARS-CoV (55.6%) and WIV1 (61.1%). In the non-severe disease group, the positivity rate for most S1 subunits ranged from 72% to 96%. However, the positivity rates for SARS-CoV S1 (22%) and WIV1 S1 (36%) were lower compared to the severe disease group. Both cohorts maintained high cross-reactivity against S2 subunits (83.3%-96%) (**Figure 1D**), consistent with the high degree of conservation observed in the S2 domain across sarbecoviruses. The breadth of S1 cross-reactivity was significantly greater in severe compared to non-severe cases, while no significant difference was observed for S2 reactivity breadth (**Figures 1B, E**).

Quantitative analysis revealed significantly higher S1-directed binding titers in severe cases for the majority of viruses tested (SARS-CoV, RaTG13, WIV1, PCoV-GX, PCoV-GD, and Civet007) (**Figure 1C**). In contrast, S2-directed titers showed minimal intergroup variation, with the exception of Civet007 (**Figure 1F**).

To compare differences in cross-neutralizing antibody levels against sarbecoviruses in plasma from recovered patients with severe versus non-severe SARS-CoV-2 infections, we constructed a sarbecovirus pseudovirus system for neutralization assays. Similar to the trend observed in cross-binding reactions, the frequency of neutralizing antibody responses against SARS-CoV was relatively low (23.53%) in plasma from recovered patients with severe infections, whereas the frequencies against the other six sarbecovirus species were higher (82.35%-94.12%). Conversely, the frequency of neutralizing antibody responses against SARS-CoV and Civet007 in plasma from recovered patients with non-severe infections was also lower, at 28% and 60%, respectively (**Figure 1G**). While the overall breadth of cross-neutralization did not differ significantly between groups (**Figure 1H**), neutralization titers against six animal-derived sarbecoviruses were consistently higher in severe convalescents (**Figure 1I**). Correlation analysis revealed positive associations between SARS-CoV-2-specific neutralization titers and cross-neutralization titers against most animal-derived sarbecoviruses (excluding SARS-CoV), suggesting that the magnitude of primary infection-induced immunity drives cross-protective responses (**Supplementary Figure 1**).

### Cross-reactive immunity demonstrates differential durability based on disease severity

**Figure 2:**
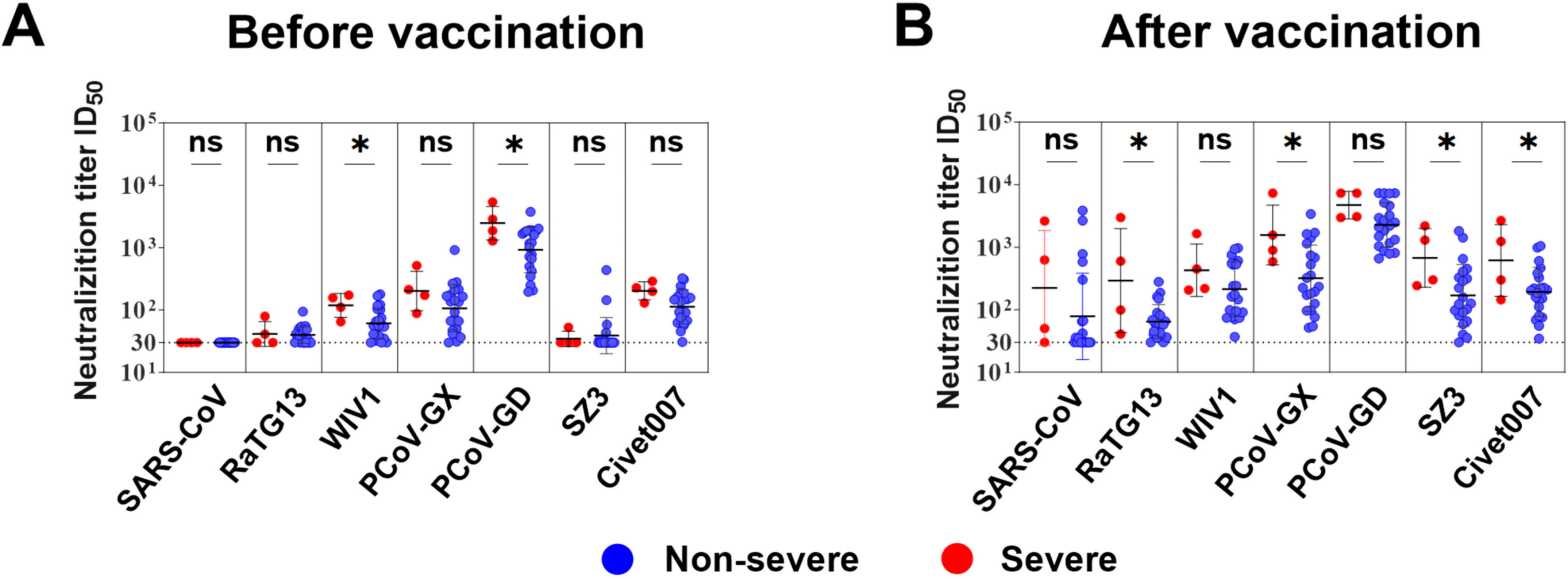
Longitudinal analysis of cross-neutralization responses. **(A)** Neutralization titers at 12 months post-infection (severe: n=4; non-severe: n=21). **(B)** Neutralization titers post-vaccination in the same cohort (severe: n=4; non-severe: n=21). Statistical comparisons were performed using the Mann-Whitney U test. *P< 0.05; **P< 0.01; ***P< 0.001; ****P< 0.0001. ns, not significant.

To evaluate the durability of cross-reactive immunity, we analyzed plasma samples from 25 convalescents at one-year post-infection (severe: n=4; non-severe: n=21). At this time point, detection showed no difference in antibody titers against S1/S2 protein binding between the two groups, with no significant differences in binding affinity observed (**Supplementary Figures 2A–D**). Neutralization titers trended higher in severe cases for most tested viruses, with statistically significant differences noted for WIV1 and PCoV-GD (**Figure 2A**).

Following vaccination, cross-reactive responses were further augmented (**Figure 2B**). While binding titers and affinity did not differ significantly between groups post-vaccination (**Supplementary Figures 3A–D**), neutralization titers were significantly elevated in severe convalescents against all tested sarbecoviruses, with increases for RaTG13, PCoV-GX, SZ3, and Civet007 reaching statistical significance (**Figure 2B**).

### Isolation and characterization of dual-targeting monoclonal antibodies

Given the importance of broad-spectrum immunity, we sought to isolate antibodies capable of simultaneously recognizing both S1 and S2 subunits. We labeled SARS-CoV-2 S1 and S2 proteins with distinct fluorescent dyes (Alexa Fluor 488 and 647, respectively) to create dual probes for memory B cell sorting. Using these probes, we employed flow cytometry to sort specific, double-positive memory B cells (phenotype: LIVE/DEAD^-^ CD3^-^ CD19^+^ CD27^+^ IgG^+^) from three convalescent vaccinees (**Supplementary Figure 4**; **Supplementary Table 3**).

**Figure 3:**
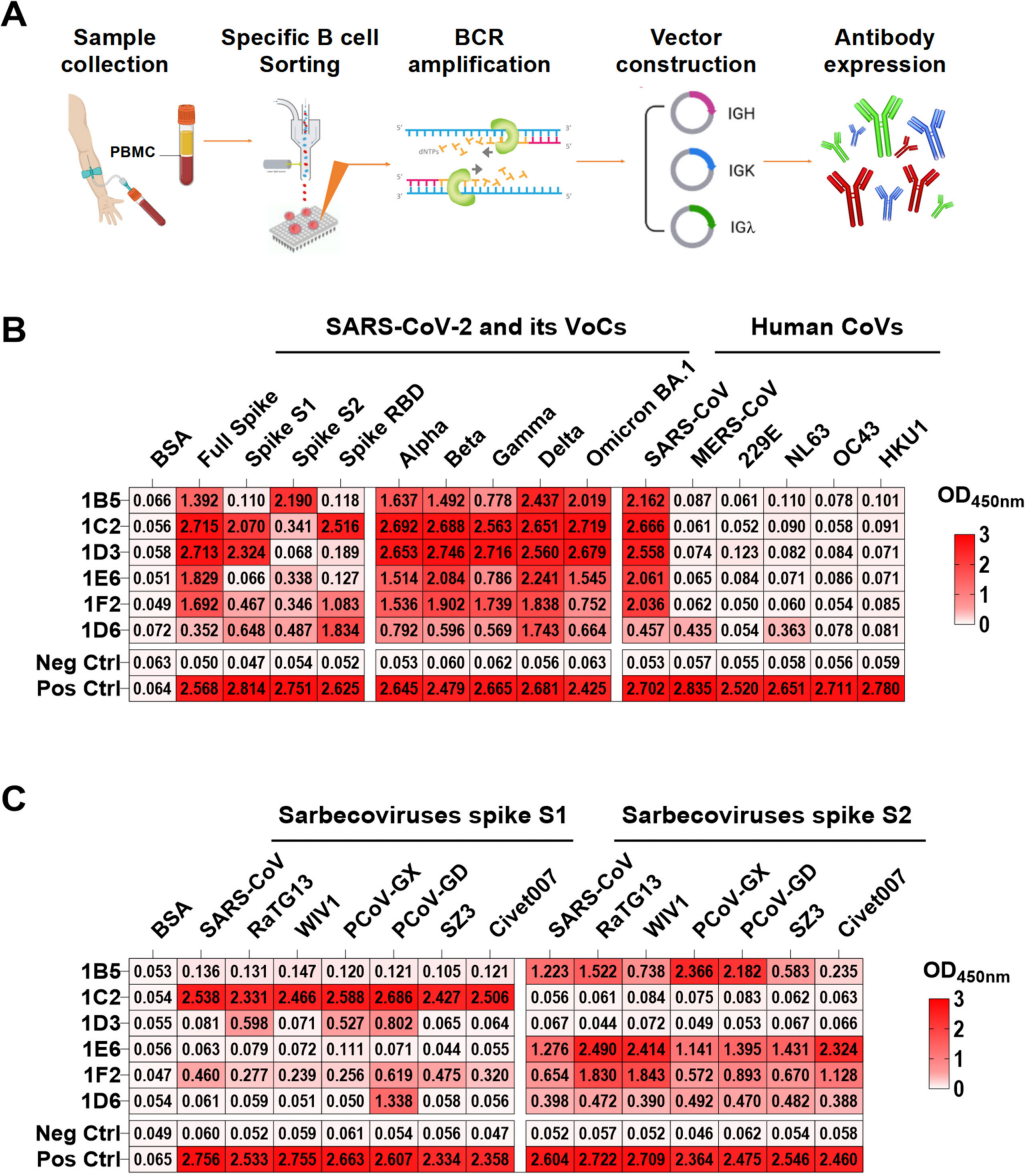
Cross-reactivity profiles of isolated monoclonal antibodies. **(A)** Workflow for mAb production by cloning antibody genes from memory B cells. **(B, C)** Cross-reactivity heatmaps showing binding of isolated monoclonal antibodies (1 μg/mL) to **(B)** SARS-CoV-2 variants of concern (VoCs) and human coronaviruses (HCoVs), and **(C)** sarbecovirus spike proteins. Color intensity represents mean optical density (OD_450_) values; samples with OD_450_ values exceeding threefold the negative control mean were classified as positive.

Single-cell sorting yielded 28 S1/S2 dual-targeting B cell clones. From these, we successfully expressed and purified six monoclonal antibodies (mAbs) with paired heavy and light chains (**Supplementary Table 4**). Binding analysis revealed distinct recognition patterns (**Figure 3A**). Three mAbs (1C2, 1F2, and 1D6) demonstrated binding to all three domains tested (S1, RBD, and S2). The remaining mAbs exhibited restricted binding profiles: 1D3 bound exclusively to S1, while 1B5 and 1E6 bound exclusively to S2. All six mAbs demonstrated binding to SARS-CoV-2 variants of concern (Alpha, Beta, Gamma, Delta, Omicron BA.1) and SARS-CoV (**Figure 3B**). Only 1D6 exhibited additional, limited cross-reactivity with other human coronaviruses (MERS-CoV and NL63) in screening assays (**Figure 3B**).

Comprehensive binding analysis of isolated antibodies against sarbecovirus spike S1 and S2 subunits derived from bats, pangolins, and civets revealed distinct patterns. Antibodies 1C2 and 1F2 exhibited broad-spectrum binding activity against seven sarbecovirus spike S1 subunits, whereas antibody 1D6 showed cross-binding activity exclusively against PCoV-GD spike S1 (**Figure 3C**). Antibodies 1B5, 1E6, 1F2, and 1D6 demonstrated broad-spectrum binding activity against seven sarbecovirus spike S2 subunits. Notably, antibody 1F2 exhibited the remarkable capacity to bind simultaneously to all seven sarbecovirus spike S1 and S2 subunits (**Figure 3C**).

### Functional characterization reveals distinct neutralization profiles

**Figure 4:**
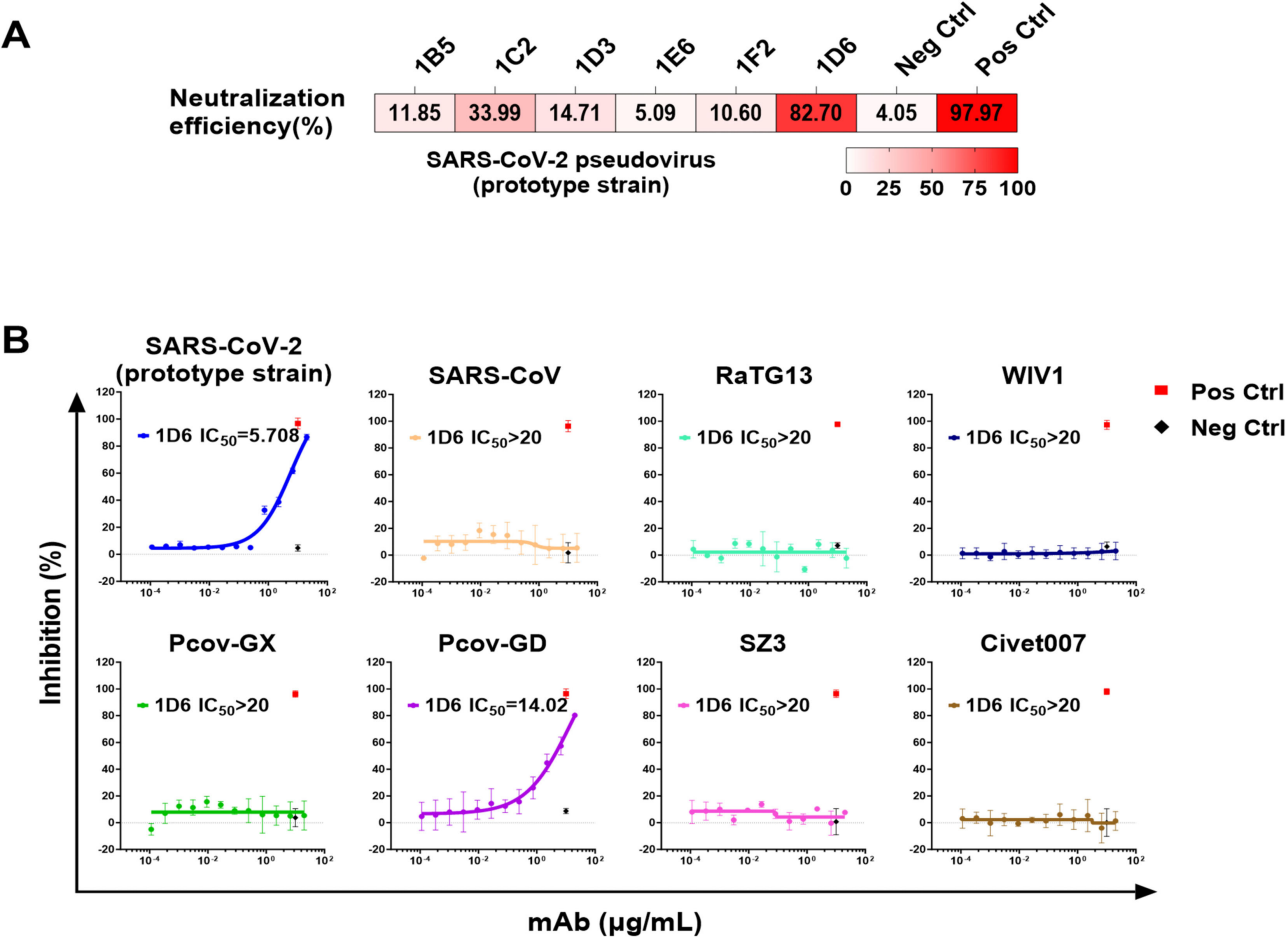
Functional assessment of neutralization capacity for isolated antibodies. **(A)** Pseudovirus neutralization assay against prototype SARS-CoV-2 performed with six monoclonal antibodies. Monoclonal antibodies demonstrating >50% neutralization at 10 μg/mL concentration were considered to exhibit neutralizing activity, with mAb SCM13–65 serving as the positive control (Pos Ctrl). **(B)** Dose-response curves and calculated IC_50_ values for mAb 1D6 against eight sarbecovirus pseudoviruses. Data represent mean ± SEM from triplicate experiments.

Neutralization screening identified mAb 1D6 as the sole clone with neutralizing activity against SARS-CoV-2 pseudovirus, while the remaining five mAbs were non-neutralizing (**Figure 4A**). Further detailed detection of 1D6’s neutralizing capacity against eight sarbecoviruses revealed that mAb 1D6 specifically neutralized SARS-CoV-2 (IC_50_ = 5.708 μg/mL) and PCoV-GD (IC_50_ = 14.02 μg/mL) pseudoviruses, but showed no activity against other tested sarbecoviruses (**Figure 4B**). This selective neutralization pattern suggests that 1D6 targets strain-specific epitopes rather than universally conserved neutralizing determinants. Competition ELISA showed that 1D6 weakly inhibited ACE2-RBD binding, with 21.05% efficiency, suggesting that its neutralization mechanism may involve partial ACE2 blocking or additional post-binding inhibitory effects (**Supplementary Figure 5**). In contrast, 1F2 failed to block ACE2-RBD interaction (6.01% inhibition), consistent with its lack of neutralization activity (**Supplementary Figure 5**).

### Structural modeling elucidates distinct binding architectures

**Figure 5:**
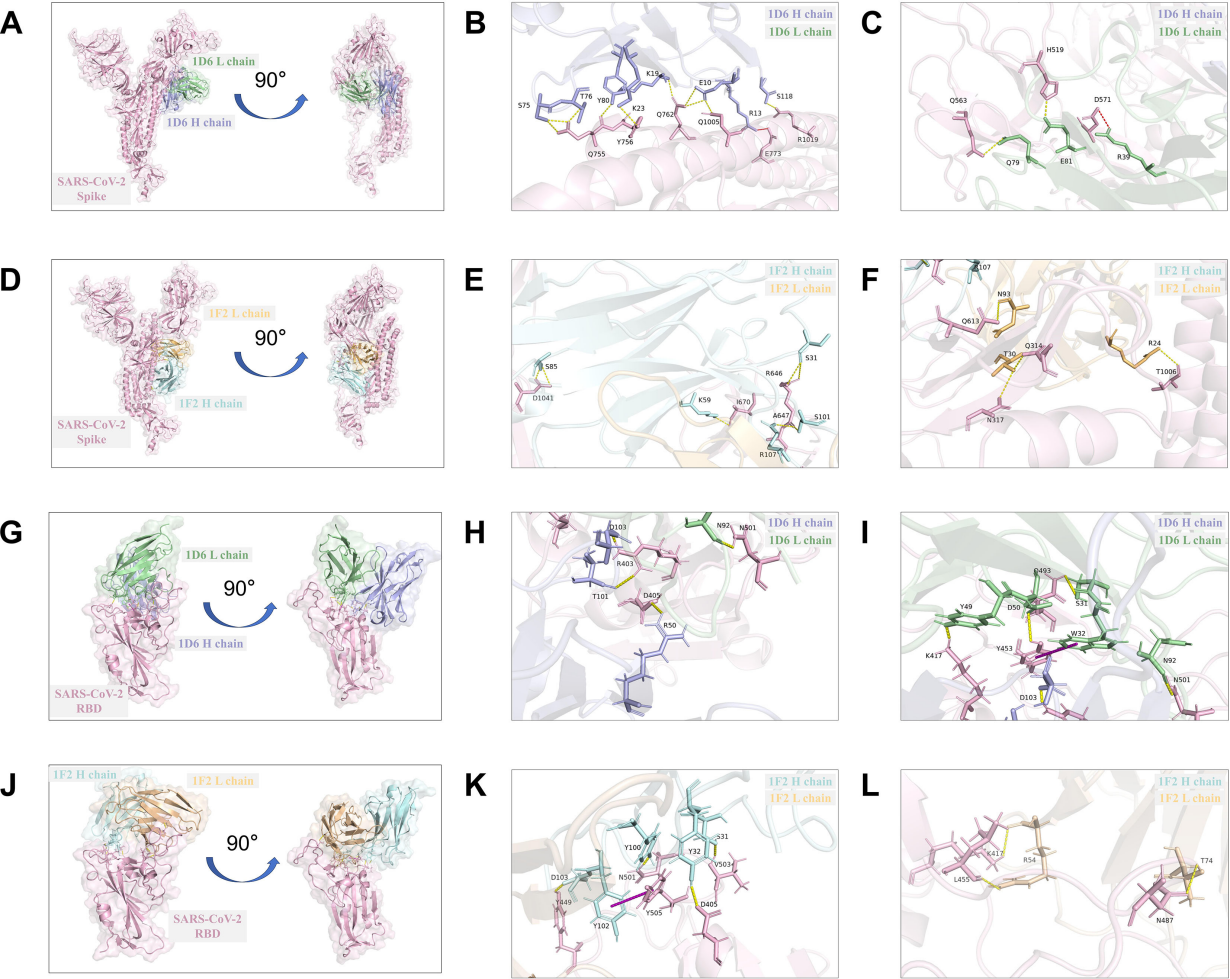
Structural characterization of dual-targeting antibody-spike interactions. **(A–C)** 1D6 binding to SARS-CoV-2 spike protein. **(A)** Overall complex structure showing 1D6 (heavy chain in blue, light chain in green) bound to spike trimer. **(B, C)** Detailed interface mapping of heavy chain **(B)** and light chain **(C)** contacts with spike protein. Critical interacting residues are annotated, with intermolecular interactions color-coded: hydrogen bonds (yellow dashed lines), hydrophobic contacts (gray), carbon-hydrogen bonds (red), and π-π stacking interactions (purple). **(D–F)** 1F2 binding to SARS-CoV-2 spike protein. **(D)** Overall complex structure showing 1F2 (heavy chain in cyan, light chain in orange) bound to spike trimer. **(E, F)** Detailed interface mapping of heavy chain **(E)** and light chain **(F)** contacts with spike protein, with annotations as described above. **(G–I)** Structural analysis of 1D6 binding to SARS-CoV-2 RBD. **(G)** Overall 1D6-RBD complex architecture. **(H, I)** Detailed heavy chain **(H)** and light chain **(I)** interaction footprints with RBD, highlighting specific recognition motifs and binding energetics. **(J–L)** Structural analysis of 1F2 binding to SARS-CoV-2 RBD. **(J)** Overall 1F2-RBD complex architecture. **(K, L)** Detailed heavy chain **(K)** and light chain **(L)** interaction footprints with RBD, demonstrating distinct binding topology compared to 1D6.

To understand the molecular mechanisms underlying distinct recognition patterns, we performed structural modeling using AlphaFold 3 (18). The analysis revealed fundamentally different binding architectures between 1D6 and 1F2 antibodies. 1D6 exhibited a compact, heavy-chain-centered binding interface dominated by polar and electrostatic interactions, featuring a dense hydrogen bond network involving spike residues Q755, Y756, Q762, and R1019 (**Figures 5A–C**). Critical interaction hubs Q755 and Y756 formed multiple hydrogen bonds with 1D6 heavy-chain residues, while salt bridges (D571–R39 and E773–R13) stabilized the complex.

In contrast, 1F2 adopted a more distributed binding interface with substantial contributions from both heavy and light chains (**Figures 5D–F**). The heavy chain contacted D1041, I670, R646, and A647, while the light chain formed hydrogen bonds with Q613, Q314, N317, and T1006, enabling recognition of a broader spike surface and explaining its enhanced cross-reactivity.

At the RBD level, 1D6 primarily targeted residues near the receptor-binding ridge (R403, D405, K417, Y453, Q493, N501) with prominent π-π stacking interactions between W99 (1D6-H) and Y456/Y489 (RBD) (**Figures 5G–I**). Notably, among these key contact residues, only position 417 differs between SARS-CoV-2 (K) and PCoV-GD (R), and this K-to-R substitution is conservative, preserving a positive charge. In contrast, other sarbecoviruses often exhibit non-conservative alterations at these sites (e.g., K417V), which are likely disruptive. This pattern of epitope conservation provides a structural basis for the observed cross-neutralization of SARS-CoV-2 and PCoV-GD, but not other sarbecoviruses, by 1D6 (**Figure 4B**). Conversely, 1F2 preferentially engaged ACE2-critical receptor-binding motif (RBM) residues (Y449, N501, D405, V503, Y505, K417, L455, N487), explaining its broad cross-reactivity despite lack of neutralization activity (**Figures 5J–L**).

Consistent with the monomeric nature of the docking models, these structural insights provide a preliminary framework suggesting how 1D6 might achieve dual-subunit targeting while maintaining strain-selective neutralization, whereas 1F2 might achieve broad cross-reactivity through distributed binding that appears insufficient to effectively interfere with viral entry mechanisms. Validation using trimeric spike structures or cryo-EM will be required to confirm these mechanistic interpretations.

## Discussion

Our findings suggest an association between COVID-19 disease severity and the development of cross-sarbecovirus immunity, which may provide insights for understanding protective immunity. The full-length spike-binding data from the 2-month cohort were originally reported in Zhang et al. (12); the current analysis extends these observations to cross-sarbecovirus responses at later time points. These findings contribute significantly to understanding how disease severity influences cross-protective immunity and provide proof-of-concept for dual-targeting antibodies as a broad-spectrum strategy. At 2 months post-infection, severe convalescents exhibited significantly enhanced cross-neutralization capacity relative to non-severe cases, consistent with a higher antigenic load during severe infection driving more robust B cell responses through increased germinal center activation and affinity maturation (19, 20). In the limited subset with 12-month follow-up data (n=4 severe cases), neutralization titers appeared to remain elevated in severe convalescents. However, given the small sample size, these observations are hypothesis-generating only and require validation in larger cohorts. The subsequent amplification of cross-neutralizing responses post-vaccination underscores the importance of immune memory formation and suggests that the magnitude of the primary immune response may establish a baseline for subsequent adaptive responses. Specifically, we hypothesize that severe infection elicits a more robust immunological memory that is preferentially amplified upon re-exposure to antigen, as evidenced by the significantly elevated post-vaccination neutralization titers in severe convalescents. This interpretation, however, remains speculative and requires validation in larger longitudinal cohorts. The identification of S2 subunit cross-reactivity across all cohorts supports the rationale for targeting the S2 subunit as a foundation for pan-coronavirus vaccines. The structural conservation of S2, particularly the fusion machinery, makes it an attractive target for broad-spectrum interventions. Our observation that S2-directed responses remain stable over time while S1 responses may wane provides compelling evidence for including S2 components in next-generation vaccines (21), suggesting that S2-focused immunogens could provide more durable protection against diverse sarbecovirus variants.

The isolation of dual-targeting antibodies 1D6 and 1F2 provides mechanistic insights into broad recognition strategies that extend beyond single-domain approaches. Unlike prior S2-directed antibodies such as S2P6 and 76E1, which target single conserved regions (22, 23), 1D6 and 1F2 employ a mechanistically distinct dual-domain strategy that simultaneously engages both the S1 and the conserved S2 fusion machinery. This architecture potentially raises the genetic barrier to viral escape beyond that achievable by single-domain S2 antibodies, though the modest potency of 1D6 highlights the need for additional affinity maturation. At the structural level, 1D6 primarily targets key residues near the receptor-binding ridge (R403, D405, K417, Y453, Q493, N501). Between SARS-CoV-2 and PCoV-GD, only a conservative K417R substitution exists at these positions, preserving the positive charge and explaining the selective cross-neutralization of these two viruses; in contrast, other sarbecoviruses often exhibit non-conservative substitutions (e.g., K417V) that likely disrupt the interaction interface. Conversely, the pan-sarbecovirus binding breadth of 1F2—despite its non-neutralizing phenotype—provides a basis for hypothesizing that Fc-optimization toward ADCC or CDC functionality may represent a viable path to therapeutic utility. As Fc-mediated effector mechanisms can contribute to viral clearance even in the absence of potent neutralization (24), future studies will need to characterize the effector functions of 1F2 to determine whether its broad-spectrum binding translates into protective activity. Consistent with its minimal ACE2-blocking activity (6.01% efficiency in competition assays), 1F2’s engagement of the RBD without neutralization suggests that certain highly conserved epitopes are not positioned to effectively interfere with viral entry, potentially due to conformational constraints or steric factors. It is important to emphasize that these structural models were generated using monomeric RBD/spike structures; therefore, conclusions regarding epitope accessibility, ACE2 blocking, and fusion inhibition should be interpreted with caution, as monomer-based modeling may not accurately reproduce the spatial constraints present in the native trimeric spike architecture.

Several limitations should be acknowledged. First, there were baseline differences in age, disease duration, and lymphocyte count between the severe and non-severe groups. Although multivariable regression analysis in our prior study demonstrated that disease severity remained independently associated with antibody responses after adjusting for these variables (12), residual confounding from all factors cannot be fully excluded in this observational cohort. Second, the 12-month follow-up included only four severe cases, substantially limiting statistical power; observed differences should be considered hypothesis-generating rather than conclusive, and larger longitudinal studies are required to validate durability patterns. Third, monoclonal antibodies were derived from PBMCs of only three convalescent vaccinees due to the resource-intensive nature of single B-cell sorting, so the isolated clones may not fully capture the breadth of the B-cell repertoire. Fourth, recombinant S1/S2 antigens were not formally validated for native trimeric conformation; isolated domains may not recapitulate the structural constraints of native trimeric spike, and future studies should employ trimeric spike constructs for more physiologically relevant antibody characterization. Fifth, although the modest neutralization potency of 1D6 (IC50 in the μg/mL range) and the lack of *in vivo* efficacy data preclude direct clinical translation—requiring affinity maturation, Fc engineering, and animal model validation to improve therapeutic utility and assess protective efficacy—the identification of 1D6 nevertheless demonstrates that the human immune system can generate antibodies with dual-domain (S1/S2) targeting capability, providing proof-of-concept for a dual-domain targeting strategy.

While the identified antibodies require substantial optimization before therapeutic application, the clinical implications of our findings extend beyond immediate antibody development. The association between disease severity and cross-protective immunity raises important questions about optimal vaccination strategies for different populations. For instance, individuals with non-severe initial infections may benefit from adjuvants or booster strategies designed to enhance cross-reactive responses. Additionally, our data suggest that monitoring cross-neutralization titers, rather than SARS-CoV-2-specific responses alone, may provide better correlates of protection against emerging variants. These findings carry significant implications for vaccine efficacy assessments and public health policy decisions. Moving forward, our findings support a multi-pronged approach to sarbecovirus preparedness. First, surveillance programs should monitor cross-reactive immunity in populations with varying disease histories to better understand population-level protection. Second, vaccine development should consider incorporating both variable and conserved epitopes to balance breadth and potency, potentially through mosaic or multivalent approaches. Third, future therapeutic strategies could benefit from combining antibodies with different mechanisms of action, such as pairing optimized dual-targeting neutralizers based on templates such as 1D6 with broadly binding non-neutralizers like 1F2 to maximize protective efficacy, pending *in vivo* validation. Finally, understanding the correlates of protection for cross-reactive immunity will guide the development of next-generation vaccines and therapeutics.

## Data Availability

The raw data supporting the conclusions of this article will be made available by the authors, without undue reservation.
