# Supplementary figures and images for: Severity-associated cross-reactive anti-sarbecovirus antibody responses in COVID-19 convalescents and isolation of a dual-targeting monoclonal antibody with cross-neutralizing activity
Source: Front Immunol. 2026 Jun 15;17:1839618. doi: 10.3389/fimmu.2026.1839618 (PMC13310989; doi:10.3389/fimmu.2026.1839618)

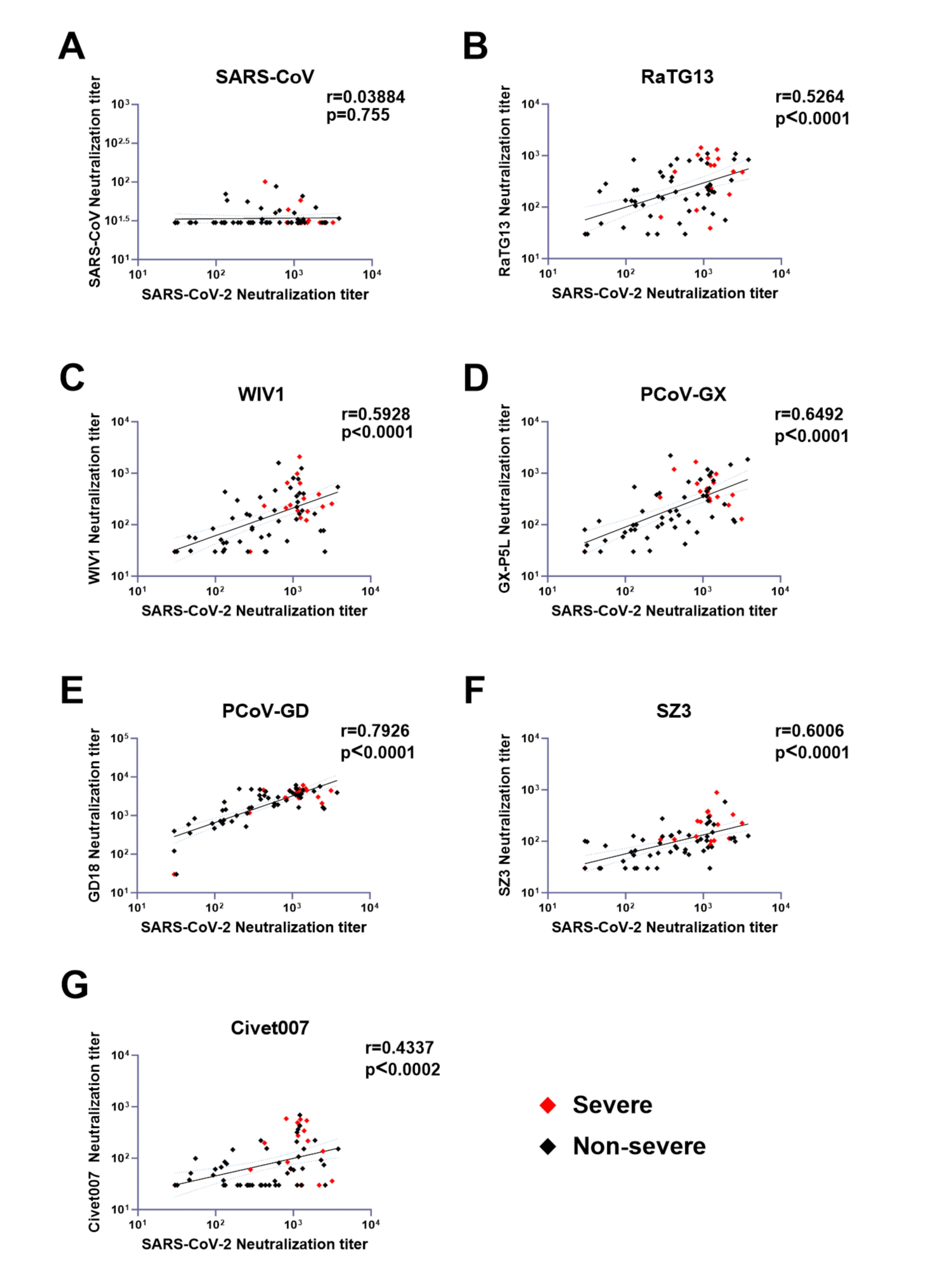

Supplement: Supplementary file 1 [file Image1.jpeg]

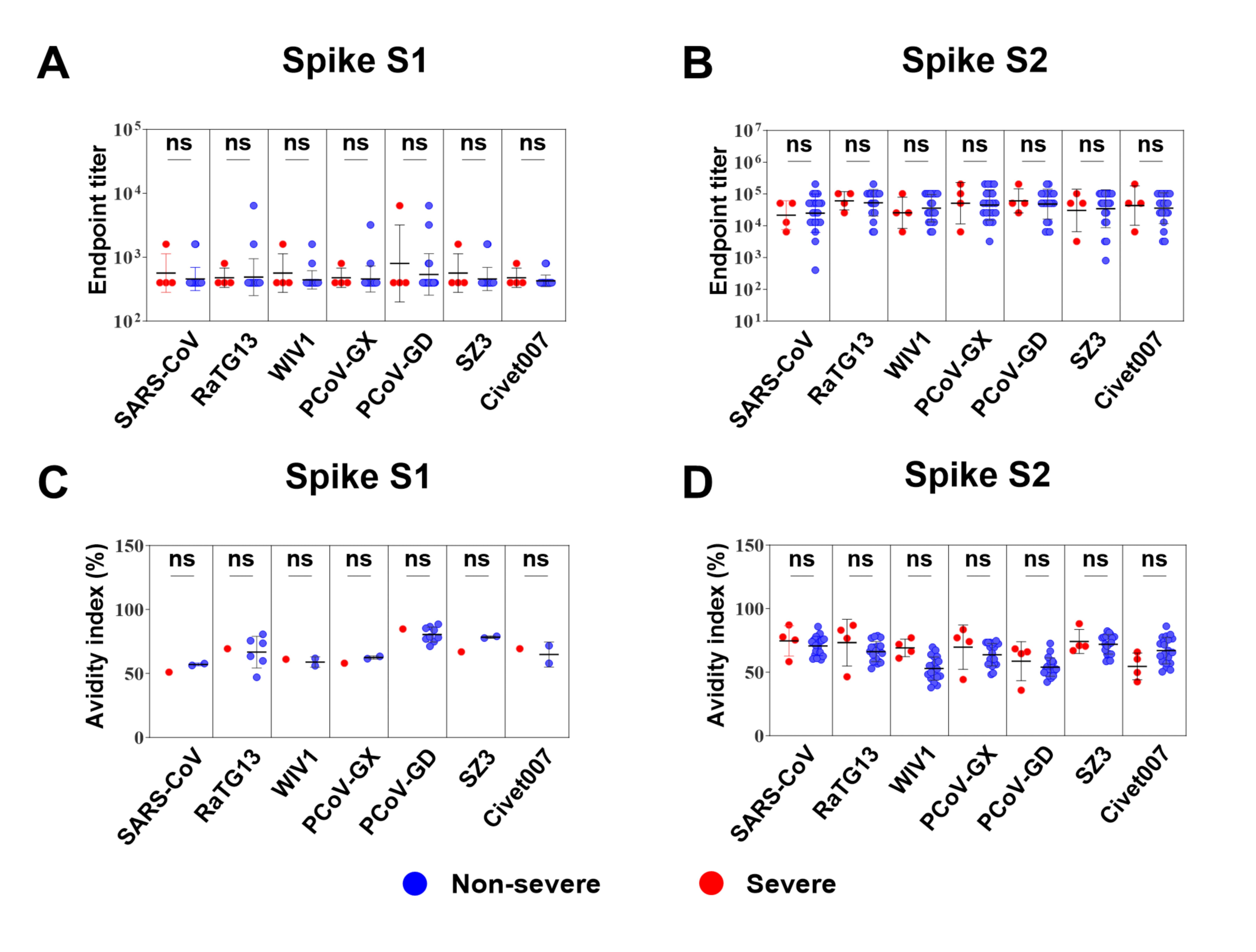

Supplement: Supplementary file 2 [file Image2.jpeg]

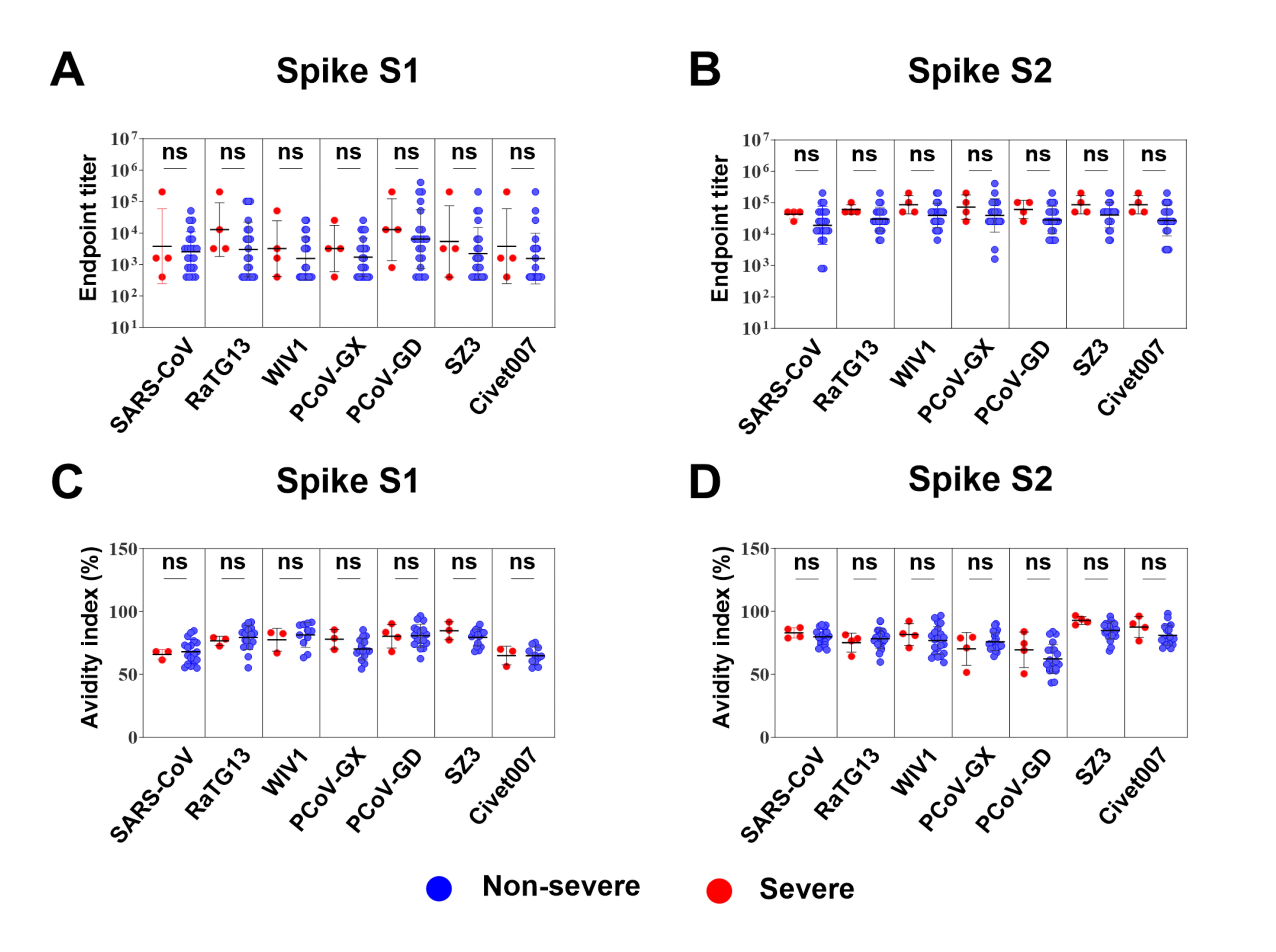

Supplement: Supplementary file 3 [file Image3.jpeg]

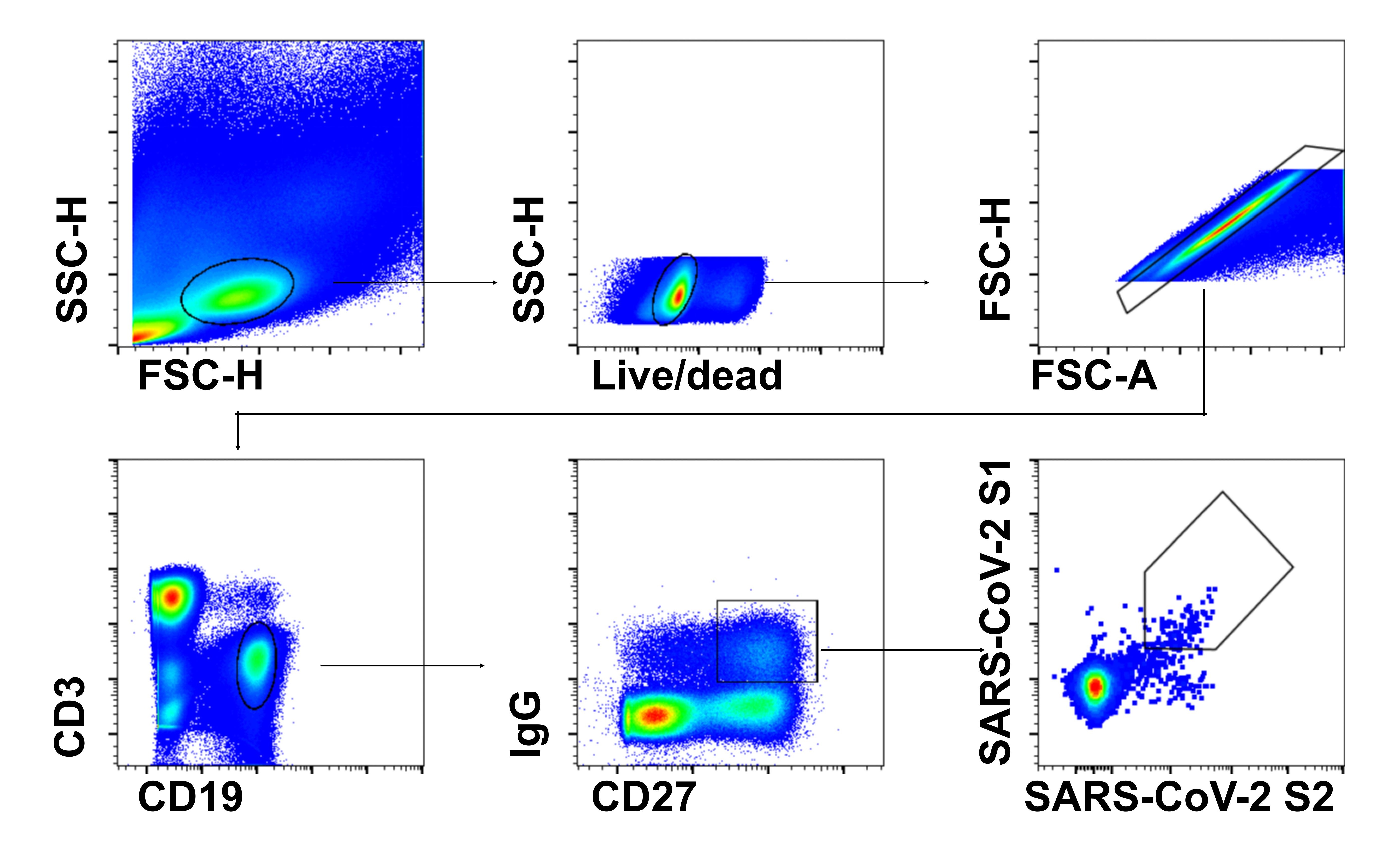

Supplement: Supplementary file 4 [file Image4.jpeg]

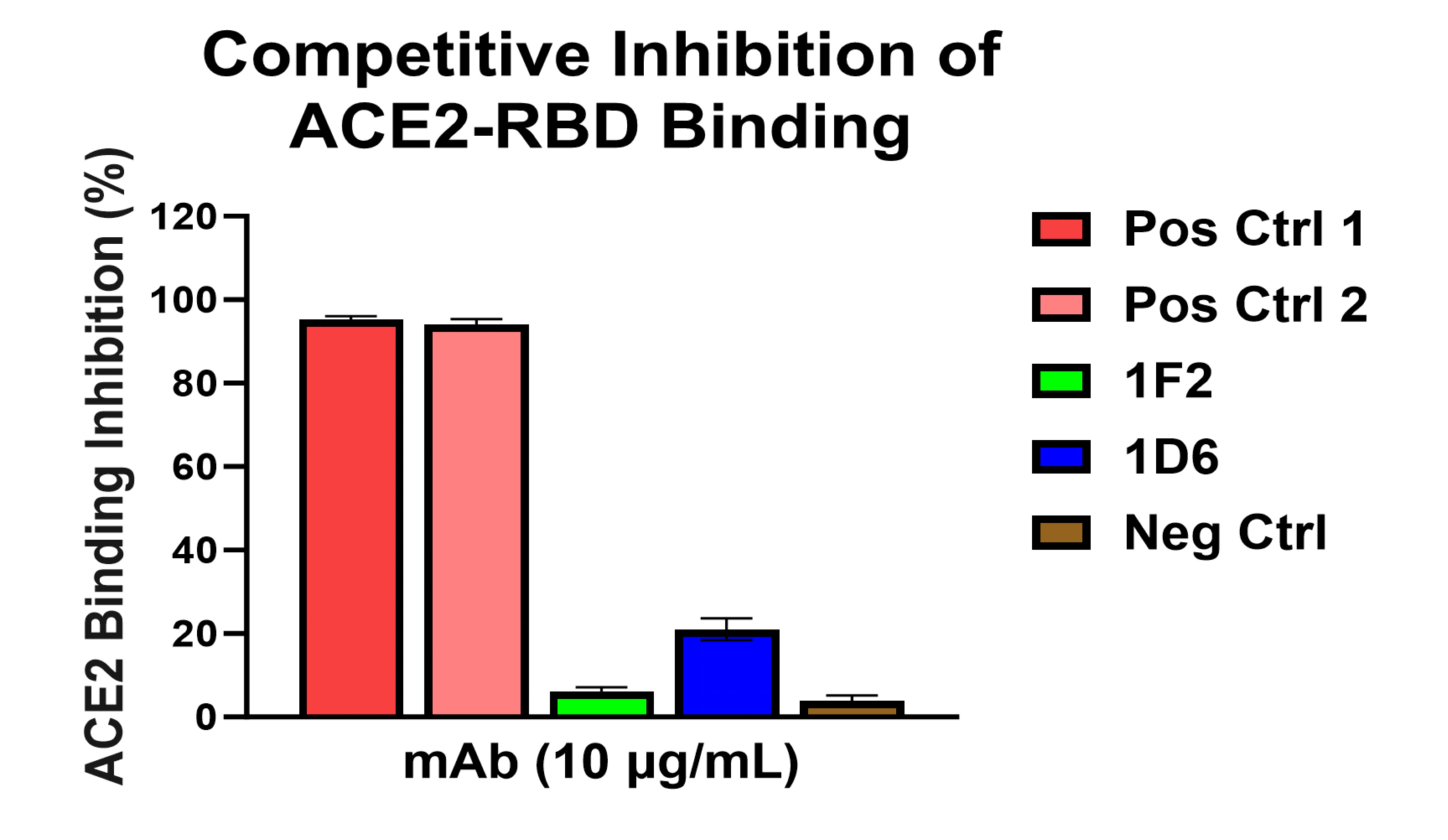

Supplement: Supplementary file 5 [file Image5.jpeg]
