# Supplementary material for: Severity-associated cross-reactive anti-sarbecovirus antibody responses in COVID-19 convalescents and isolation of a dual-targeting monoclonal antibody with cross-neutralizing activity
Source: Front Immunol. 2026 Jun 15;17:1839618. doi: 10.3389/fimmu.2026.1839618 (PMC13310989; doi:10.3389/fimmu.2026.1839618)
Supplement: Supplementary file 6 [file Table1.docx]

**Supplementary Table S1. Baseline demographic and clinical characteristics of COVID-19 convalescent participants**

| **Patient ID** | **Severity of disease** | **Age (year)** | **Sex** | **Race** | **Course of disease (day)** |
| --- | --- | --- | --- | --- | --- |
| Patient 1 | Non-severe | 29 | Female | Han Chinese | 20 |
| Patient 2 | Non-severe | 54 | Female | Han Chinese | 14 |
| Patient 3 | Non-severe | 29 | Female | Han Chinese | 30 |
| Patient 4 | Severe | 62 | Male | Han Chinese | 15 |
| Patient 5 | Severe | 84 | Female | Han Chinese | 35 |
| Patient 6 | Non-severe | 43 | Female | Han Chinese | 24 |
| Patient 7 | Severe | 43 | Male | Han Chinese | 21 |
| Patient 8 | Severe | 43 | Male | Han Chinese | 13 |
| Patient 9 | Non-severe | 43 | Female | Han Chinese | 12 |
| Patient 10 | Non-severe | 39 | Female | Han Chinese | 19 |
| Patient 11 | Non-severe | 23 | Male | Han Chinese | 19 |
| Patient 12 | Severe | 62 | Male | Han Chinese | 17 |
| Patient 13 | Non-severe | 28 | Female | Han Chinese | 28 |
| Patient 14 | Non-severe | 36 | Male | Han Chinese | 18 |
| Patient 15 | Non-severe | 29 | Male | Han Chinese | 16 |
| Patient 16 | Severe | 82 | Female | Han Chinese | 22 |
| Patient 17 | Non-severe | 53 | Female | Han Chinese | 29 |
| Patient 18 | Non-severe | 47 | Female | Han Chinese | 45 |
| Patient 19 | Non-severe | 23 | Male | Han Chinese | 17 |
| Patient 20 | Non-severe | 47 | Male | Han Chinese | 16 |
| Patient 21 | Non-severe | 38 | Male | Han Chinese | 32 |
| Patient 22 | Non-severe | 47 | Female | Han Chinese | 24 |
| Patient 23 | Non-severe | 46 | Female | Han Chinese | 16 |
| Patient 24 | Non-severe | 45 | Female | Han Chinese | 18 |
| Patient 25 | Severe | 34 | Female | Han Chinese | 28 |
| Patient 26 | Non-severe | 48 | Male | Han Chinese | 12 |
| Patient 27 | Non-severe | 20 | Male | Han Chinese | 13 |
| Patient 28 | Severe | 47 | Male | Han Chinese | 21 |
| Patient 29 | Non-severe | 22 | Male | Han Chinese | 14 |
| Patient 30 | Non-severe | 39 | Female | Han Chinese | 23 |
| Patient 31 | Non-severe | 40 | Male | Han Chinese | 20 |
| Patient 32 | Non-severe | 33 | Male | Han Chinese | 17 |
| Patient 33 | Non-severe | 62 | Male | Han Chinese | 22 |
| Patient 34 | Non-severe | 50 | Female | Han Chinese | 23 |
| Patient 35 | Non-severe | 27 | Male | Han Chinese | 14 |
| Patient 36 | Non-severe | 23 | Male | Han Chinese | 23 |
| Patient 37 | Non-severe | 43 | Male | Han Chinese | 25 |
| Patient 38 | Non-severe | 50 | Female | Han Chinese | 25 |
| Patient 39 | Non-severe | 45 | Female | Han Chinese | 25 |
| Patient 40 | Non-severe | 43 | Male | Han Chinese | 27 |
| Patient 41 | Severe | 59 | Female | Han Chinese | 28 |
| Patient 42 | Severe | 33 | Male | Han Chinese | 27 |
| Patient 43 | Non-severe | 23 | Male | Han Chinese | 16 |
| Patient 44 | Non-severe | 41 | Male | Han Chinese | 23 |
| Patient 45 | Severe | 67 | Female | Han Chinese | 25 |
| Patient 46 | Non-severe | 30 | Female | Han Chinese | 18 |
| Patient 47 | Severe | 72 | Female | Han Chinese | 22 |
| Patient 48 | Non-severe | 30 | Female | Han Chinese | 17 |
| Patient 49 | Non-severe | 50 | Female | Han Chinese | 17 |
| Patient 50 | Non-severe | 47 | Male | Han Chinese | 17 |
| Patient 51 | Non-severe | 22 | Male | Han Chinese | 18 |
| Patient 52 | Severe | 54 | Male | Han Chinese | 28 |
| Patient 53 | Non-severe | 46 | Female | Han Chinese | 11 |
| Patient 54 | Severe | 53 | Male | Han Chinese | 24 |
| Patient 55 | Non-severe | 41 | Female | Han Chinese | 24 |
| Patient 56 | Non-severe | 20 | Female | Han Chinese | 15 |
| Patient 57 | Non-severe | 69 | Female | Han Chinese | 18 |
| Patient 58 | Non-severe | 45 | Male | Han Chinese | 21 |
| Patient 59 | Severe | 81 | Male | Han Chinese | 30 |
| Patient 60 | Non-severe | 51 | Female | Han Chinese | 15 |
| Patient 61 | Non-severe | 21 | Female | Han Chinese | 22 |
| Patient 62 | Severe | 53 | Male | Han Chinese | 29 |
| Patient 63 | Non-severe | 82 | Male | Han Chinese | 20 |
| Patient 64 | Severe | 64 | Male | Han Chinese | 27 |
| Patient 65 | Non-severe | 29 | Male | Han Chinese | 12 |
| Patient 66 | Non-severe | 32 | Female | Han Chinese | 16 |
| Patient 67 | Non-severe | 41 | Female | Han Chinese | 16 |
