# Supplementary material for: Severity-associated cross-reactive anti-sarbecovirus antibody responses in COVID-19 convalescents and isolation of a dual-targeting monoclonal antibody with cross-neutralizing activity
Source: Front Immunol. 2026 Jun 15;17:1839618. doi: 10.3389/fimmu.2026.1839618 (PMC13310989; doi:10.3389/fimmu.2026.1839618)
Supplement: Supplementary file 7 [file Table2.docx]

**Supplementary Table S2. Baseline characteristics of enrolled COVID-19 convalescents pre- and post-vaccination**

| **Patient ID** | **Sex** | **Severity of disease** | **Age (year)** | **Sample collection time (day after illness onset)** | **Sample collection time (day after third dose administration)** |
| --- | --- | --- | --- | --- | --- |
| Patient 1 | Male | Non-severe | 29 | 254 | 123 |
| Patient 2 | Male | Non-severe | 48 | 248 | 155 |
| Patient 3 | Male | Severe | 47 | 387 | 74 |
| Patient 4 | Female | Non-severe | 29 | 382 | 201 |
| Patient 5 | Female | Non-severe | 20 | 376 | 165 |
| Patient 6 | Female | Non-severe | 50 | 382 | 149 |
| Patient 7 | Female | Non-severe | 46 | 371 | 144 |
| Patient 8 | Male | Non-severe | 43 | 375 | 139 |
| Patient 9 | Female | Non-severe | 43 | 382 | 15 |
| Patient 10 | Female | Non-severe | 45 | 386 | 166 |
| Patient 11 | Male | Severe | 54 | 378 | 118 |
| Patient 12 | Male | Non-severe | 43 | 378 | 151 |
| Patient 13 | Female | Severe | 72 | 367 | 150 |
| Patient 14 | Male | Non-severe | 47 | 368 | 15 |
| Patient 15 | Male | Non-severe | 45 | 382 | 21 |
| Patient 16 | Female | Non-severe | 45 | 382 | 21 |
| Patient 17 | Female | Non-severe | 51 | 377 | 22 |
| Patient 18 | Female | Non-severe | 69 | 375 | 40 |
| Patient 19 | Female | Non-severe | 47 | 382 | 168 |
| Patient 20 | Male | Severe | 53 | 382 | 21 |
| Patient 21 | Male | Non-severe | 62 | 375 | 15 |
| Patient 22 | Male | Non-severe | 26 | 371 | 155 |
| Patient 23 | Male | Non-severe | 43 | 376 | 40 |
| Patient 24 | Male | Non-severe | 75 | 379 | 40 |
| Patient 25 | Female | Non-severe | 19 | 369 | 26 |
