# Supplementary material for: Severity-associated cross-reactive anti-sarbecovirus antibody responses in COVID-19 convalescents and isolation of a dual-targeting monoclonal antibody with cross-neutralizing activity
Source: Front Immunol. 2026 Jun 15;17:1839618. doi: 10.3389/fimmu.2026.1839618 (PMC13310989; doi:10.3389/fimmu.2026.1839618)
Supplement: Supplementary file 8 [file Table3.docx]

**Supplementary Table S3. Donor characteristics for antibody cloning**

| **Patient ID** | **Sex** | **Severity of disease** | **Age (year)** | **Sample collection time (day after third dose vaccine administration)** | **Antibodies isolated** |
| --- | --- | --- | --- | --- | --- |
| **Patient 1** | Male | Non-severe | 29 | 123 | 1C2, 1B5 |
| **Patient 2** | Male | Severe | 47 | 74 | 1F2, 1D3, 1E6 |
| **Patient 3** | Female | Non-severe | 50 | 149 | 1D6 |
