# Supplementary material for: Severity-associated cross-reactive anti-sarbecovirus antibody responses in COVID-19 convalescents and isolation of a dual-targeting monoclonal antibody with cross-neutralizing activity
Source: Front Immunol. 2026 Jun 15;17:1839618. doi: 10.3389/fimmu.2026.1839618 (PMC13310989; doi:10.3389/fimmu.2026.1839618)
Supplement: Supplementary file 9 [file Table4.docx]

**Supplementary Table S4. The characteristics of gene usage, somatic mutations, and CDR3 length of six mAbs.**

| **mAbs** | **1B5** | **1C2** | **1D3** | **1E6** | **1F2** | **1D6** |
| --- | --- | --- | --- | --- | --- | --- |
| **H-V gene** | IGHV4-59 | IGHV1-46 | IGHV4-4 | IGHV4-39 | IGHV4-59 | IGHV3-66 |
| **H-D gene** | IGHD5-18 | N/A | IGHD3-22 | IGHD3-10 | IGHD6-6 | IGHD1-26 |
| **H-J gene** | IGHJ3*02 | IGHJ6*03 | IGHJ4*02 | IGHJ3*01 | IGHJ4*02 | IGHJ2*01 |
| **H-Identity to germline (%)** | 96.6 | 93.2 | 96.9 | 96.2 | 95.5 | 91.5 |
| **H-CDR3 length** | 16 | 13 | 20 | 20 | 16 | 10 |
| **L-V gene** | IGKV1-39 | IGKV1-39 | IGKV1-39 | IGLV2-14 | IGKV3-11 | IGLV1-40 |
| **L-J gene** | IGKJ5*01 | IGKJ3*01 | IGKJ2*01 | IGLJ2*01 | IGKJ4*01 | IGLJ1*01 |
| **L-Identity to germline (%)** | 98.9 | 93.9 | 97.5 | 96.6 | 94.4 | 96.2 |
| **L-CDR3 length** | 8 | 10 | 10 | 10 | 11 | 11 |
